# Presidential address: demonstration of the international leadership of the Korea Health Personnel Licensing Examination Institute, introduction of a clinical skills test to the Korean dental licensing examination, and strengthening of ethics items on licensing examinations

**DOI:** 10.3352/jeehp.2019.16.15

**Published:** 2019-06-05

**Authors:** Yoon-Seong Lee

**Affiliations:** President, Korea Health Personnel Licensing Examination Institute, Seoul, Korea; Hallym University, Korea

The Korea Health Personnel Licensing Examination Institute (KHPLEI), which succeeded the National Health Personnel Licensing Examination Board of Korea, was launched as a government-supported special foundation in 2015 [1]. Starting on April 22, 2019, I will serve as the 8th president of the institute for the next 3 years, with the goal of proactively responding to changing social needs and further improving the process of licensing examinations. By bolstering the evidence base of the national licensing examination policies of the 26 health professions within the purview of the KHPLEI and improving the quality of the evaluations, the KHPLEI will seek to contribute to public health and well-being by licensing only the most qualified healthcare and medical professionals.

To achieve those goals, the KHPLEI will first cultivate experts specializing in the development of high-quality test items to improve the overall quality of the evaluations. The test construction center of the KHPLEI at Chungju will operate year-round to select items and train a pool of experts for advanced item development [2].

Second, the role of the research and development headquarters of KHPLEI will be reinforced; furthermore, all possible resources will be provided to support internal research efforts. Doctorate-level researchers will be trained within the institute, and simultaneous collaboration with external experts will be continued. Furthermore, all available test results, excluding any personal information, will be made accessible for research. Such efforts will receive special emphasis because the KHPLEI is internationally known as the leading research institute on healthcare and medical professional licensing examinations.

Third, services provided to examinees will be improved to make the test experience more convenient. Following international trends, the KHPLEI has successfully implemented a computer-based testing for licensing emergency medicine technicians. Similar steps will be taken for other licensing examinations to more accurately assess examinees’ competency. Legal means to prevent the leakage of test items will be sought to further streamline the implementation of computer-based testing or computerized adaptive testing [2].

Fourth, the KHPLEI will export its expertise in systems management, which has been accumulated through extensive experience, to aid foreign countries in their management of licensure. On March 14, 2019, the Korean Ministry of Health and its Vietnamese counterpart signed a memorandum of understanding (MOU) for managing licensing examinations at the InterContinental Grand Seoul Parnas Hotel (Fig. 1). This MOU intends to aid the Ministry of Health of Vietnam, and contains statements regarding the implementation and management of licensing examinations (e.g., establishing standards for item development via job analysis, advance preparation before test implementation, development and management of test items, test implementation, and management after licensing). These supportive efforts will expand to other Asian countries and beyond.

Recently, the institute has focused on the implementation of a clinical skills examination as part of the Korean dental licensing examination, which will take effect in the second half of 2021. Using dental mannikins and standardized patients, the clinical skills exam will assess candidates’ clinical performance and competency in basic skills. Moreover, the KHPLEI will enhance candidates’ clinical competency by assessing their communication skills with patients. As an international parallel, a non-patient-based clinical licensure examination for dentistry was initially implemented along with a patient-based clinical licensure examination by the state of Minnesota’s Board of Dentistry in 2009; however, the board discontinued the patient-based examination in 2018 [3]. The KHPLEI plans to only implement a non-patient-based clinical licensure examination and to utilize standardized patients for the assessment of communication skills.

Furthermore, the clinical portion of the Korean medical licensing examination, which was implemented in 2010, will be reviewed and refined. The examination has maintained an adequate level of difficulty, with an overall pass rate of 95%-97% for the last 10 years. The revised examination will reflect real-world medicine more closely by incorporating current ideas on clinical reasoning and testing examinees’ competency in establishing a positive doctor-patient relationship. Further research will also be conducted on topics such as altering the number of items (currently 12, including 6 objective structured clinical examination [OSCE] items and 6 clinical performance test questions), structuring the clinical items in the OSCE, whether to maintain inter-stationary examinations, assessing examinees’ ability to complete medical records, and improving the standard-setting method. The final decision on whether to implement these measures in 2021 will be made soon.

Additionally, new approaches to improve the inclusion of ethics items in healthcare and medical licensing examinations will be reviewed. To promote comprehensive training in ethics, the KHPLEI will develop more relevant items on ethics and incorporate them in national examinations.

The *Journal of Educational Evaluation for Health Professions* founded in 2004 has successfully published 305 articles over the 16 years. The journal now enjoys an international reputation as a renowned academic journal, as it was listed in Emerging Sources Citation Index in December 2015, in MEDLINE in March 2016,in Embase in 2018, in Scopus in January 2019, and in the Korea Citation Index (the Korea Research Foundation’s database for abstracts published in Korea) in April 2019 [4]. Because these astounding achievements were made possible by the efforts of the editors and reviewers who actively participated in this endeavor, I would like to express my greatest appreciation for them. I wish the best of health and happiness to all researchers and readers who visit the *Journal of Educational Evaluation for Health Professions*.

## Figures and Tables

**Fig. 1.**
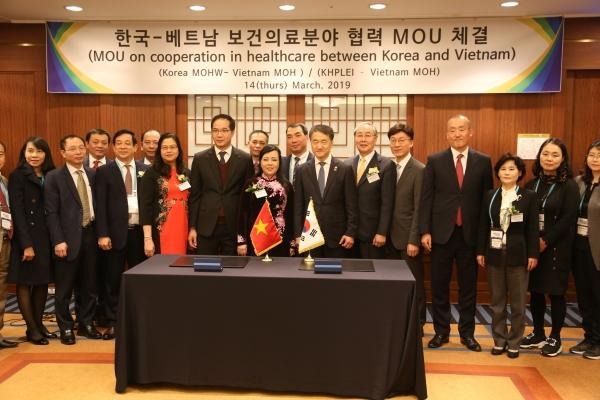
Photo of the memorandum of understanding (MOU) signing ceremony between the Ministry of Health of Vietnam and the Ministry of Health and Welfare of Korea held on March 14, 2019 at the InterContinental Grand Seoul Parnas Hotel, Seoul, Korea.

## Supplementary materials

Supplement 1. Audio recording of the text.
